# Access to Healthcare Among Young Adult Probationers Participating in a Pilot Health-Focused Reentry Program

**DOI:** 10.1177/0306624X241240700

**Published:** 2024-03-25

**Authors:** Victoria D. Ojeda, Tamara Parker, Maurice Lyles, Todd M. Edwards, Cielo Jimenez, Sarah Hiller-Venegas, Emily Berliant, Zephon Lister

**Affiliations:** 1 University of California, San Diego, La Jolla, USA; 2 University of California, San Diego School of Medicine, La Jolla, USA

**Keywords:** reentry, probationer, young adult, Health Coaching, Service Navigation, access to care, health insurance, usual source of care

## Abstract

Justice-involved adults experience disparities in healthcare access. This pilot study examines healthcare access among young adult probationers (*n* = 66) receiving 6-months of Service Navigation and Health Coaching support implemented between 2017 and 2021. Data are from baseline, 6-month follow-up and satisfaction surveys. Between baseline and follow-up, the proportion of insured young adult participants (66%–88%; *p* < .001) and those using healthcare services (36%–71%; *p* < .001) increased significantly; report of unmet physical healthcare needs decreased significantly (44%–26%; *p* = .003). Satisfaction data revealed increased self-efficacy, motivation, focus, and improved organizational, goal setting, and communication skills. The program improved healthcare access by increasing health insurance and recent use of healthcare services. Longitudinal studies are needed to assess maintenance of these outcomes and potential impacts on disparities in health status and access to care indicators. Integrating navigation and coaching supports to advance the well-being of justice-involved young adults is a promising mechanism to facilitate healthcare access.

## Introduction

In the U.S., presently ~157,000 persons are incarcerated in prisons and ~6% are ages 18 to 26; overall, Black individuals (39%) and males (93%) are disproportionately incarcerated (Federal Bureau of Prisons, 2023). In 2021, 636,300 persons were detained in U.S. jails, and 17% were ages 18 to 24; racial/ethnic minorities are also over-represented among persons in jail (Zeng, 2022). Most persons in prisons and jail return to their communities and young adult reentrants (i.e., ages 18–26 who have exited carceral settings) have emerged as a priority population within public health and criminal justice spheres as there is an increased recognition that young adult reentrants are in a state of pervasive biological, social and emotional maturation, thus reentry services should be tailored to their needs (California Department of Health Care Services, 2023b; Chung & Hudziak, 2017; Moe Urrea, 2022).

Justice-involved adults (JIA) face disparities in their access to healthcare and they are more likely than non-JIA to exhibit health conditions that require regular monitoring and care (Hawks et al., 2020). Healthcare access is defined as including health insurance coverage to overcome financial barriers, timely use of services, including for screening and prevention, and having a workforce that can meet the needs of patients (Agency for Health Research and Quality, 2022). A recent study analyzed data from four waves of the U.S. National Survey on Drug use and Health and identified non-elderly adult probationers (ages 18–49) as having a higher burden of multiple physical and mental health conditions and substance use disorder versus the general population. Moreover, a higher proportion of probationers were uninsured, living in poverty, using emergency department services, or were hospitalized as compared to the general population (Hawks et al., 2020). Recent analyses of U.S. Bureau of Justice Statistics further confirmed a higher burden of disease among are incarcerated adults as compared to the general population, including among young adults ages 18 to 33, suggesting that interventions are needed to address health disparities among young adults who have been incarcerated (Nowotny et al., 2017).

Several factors are tied to disparities in reentrants’ health and healthcare access including health literacy which refers to the skills that support one’s ability to access, understand, and use resources that promote and maintain health (Batterham et al., 2016; Berkman et al., 2011; Nutbeam, 1998). Good health literacy is critical in the current healthcare landscape where patient-centered care is pervasive and individuals are expected to use health services properly, communicate effectively with providers, assess risks/benefits of care, and locate and evaluate information for quality and validity (National Institutes of Health, Network of the National Library of Medicine, 2020). To achieve these tasks, JIA must have visual, numeric and digital literacy skills, be able to interpret health information, and have strong communication skills to support decision-making with providers (Kindig et al., 2004; National Institutes of Health, Network of the National Library of Medicine, 2020). Approximately ~60% of JIA in the United States have *inadequate* health literacy (Hadden et al., 2018; Puglisi et al., 2017). These data suggest that addressing JIA’s health literacy may be an important component of interventions seeking to reduce disparities.

Societal factors may also negatively impact JIA’s well-being. For example, JIA often experience overlapping stigmatized identities and discrimination (e.g., justice-involved status, racial and ethnic minority status, low education and income, unstably housed, living with mental illness, substance use-related; Biancarelli et al., 2019; Motavalli et al., 2021). Financial barriers can result in delaying or forgoing use of healthcare; notably, about 26% of JIA are uninsured (Hawks et al., 2020). Stigma and discrimination in healthcare settings may also deter JIA’s healthcare use, impede disclosure of harmful behaviors, or result in sub-optimal delivery of healthcare (Biancarelli et al., 2019; Hadden et al., 2018; Motavalli et al., 2021). *Whole-person interventions* that eliminate social and financial barriers to healthcare and basic needs (e.g., jobs, housing, food) *and* improve health literacy among JIA are urgently needed.

Peer specialists are increasingly delivering programs that serve JIA and subgroups (e.g., justice-involved Veterans; Hyde et al., 2022). For example, the Transitions Clinic Network approach involves training and hiring community health workers with lived incarceration experience to engage and support reentrants in primary care settings (Wang et al., 2012). Reentrants often receive services for specific needs. For example, persons living with opioid use disorder or HIV are often linked to post-release methadone maintenance or buprenorphine/naloxone treatment; antiretroviral therapy, respectively (Malta et al., 2019; Rowell-Cunsolo & Hu, 2020). Pre-release planning, including preparing to support reentrants’ health and healthcare access, is recognized as a critical element of the reentry process yet many communities face challenges in supporting individuals who are returning to the community (Ellis & Henderson, 2016).

Alternatively, patient-centered strategies to build health and health literacy include Health Coaching and Service Navigation; both can be implemented by clinicians or peer educators (Ghorob et al., 2013; Logan et al., 2015). Health Coaching can help individuals manage chronic conditions and improve medication adherence and is associated with improved shared decision-making, information sharing, behavior change (Ghorob et al., 2013) and reduced inpatient and total healthcare costs (Jonk et al., 2015). Service Navigation support may be offered individually or in cross-disciplinary teams. Navigators can help patients overcome barriers to healthcare use and underlying social determinants of health (e.g., insurance coverage, housing, employment, nutritional support) or support patient decision-making, provide social support as individuals face health or social transitions. Both Health Coaching and Service Navigation can help individuals develop or improve executive functioning skills needed to manage one’s health well-being throughout the reentry process (e.g., goal-setting, time management, resource procurement; Ojeda et al., 2022).

Taken together, the aforementioned data have fostered an interest in exploring whether health-focused reentry services can address the underlying factors that contribute to disparities in health and healthcare access among young adult reentrants (U.S. Department of Health and Human Services Office of Minority Health, 2021). Specifically, despite the separate use of Health Coaching and Service Navigation models, our research team is unaware of interventions that have integrated both modalities with the goal of improving healthcare access among justice-involved young adults; this study addresses this gap. We use the systems-level Gelberg-Andersen Behavioral Model of Service Utilization to guide the analyses and consider the role of predisposing demographic factors and enabling variables (e.g., income, housing, food security, transportation) vis-à-vis healthcare utilization. Need variables include self-identified health (Aday, 1993; Andersen, 1995; Gelberg et al., 2000; Kulkarni et al., 2010; Phillips et al., 1998; Stein et al., 2007).

Specifically, this study examines whether healthcare access changes between baseline and 6-month follow-up among a sample of young adults ages 18 to 26 who exited jail, were on probation, and participated in a pilot 6-month health-focused reentry program implemented in a large Southern California County. For this study, access to care is defined by individuals’ current health insurance status, having a regular source of care, and having a recent (i.e., past 6-months) doctor visit. Additionally, we explore participants’ perceptions of this novel health-focused reentry program through analyses of text satisfaction data.

## Methods

### Intervention Description

In brief, the UCSD Re-Entry Community Linkages (RELINK) program was a pilot health-focused reentry program for young adults ages 18 to 26 who exited jail and were on probation in San Diego, California; participation was voluntary. Recruitment was held weekly at probation offices; eligible and interested persons were enrolled and written informed consent was obtained. The University’s Human Subjects Research Protections Program approved the study. The program was implemented between February 2017 to June 2021.

The program adopted a team-based, collaborative care approach (Baik, 2017) and strived to meet participants “where they are at” as described below. Participants received one-on-one Service Navigation and an optional Health Coaching curriculum which was identified as a “Health Fellowship.” The weekly program was delivered over 6-months. Service Navigators were trained community members who provided case management and emotional, instrumental, and informational support through mentorship, modeling of pro-social behaviors, and warm handoffs to health, social service, education, and employment providers. Service Navigators also used didactic program materials addressing life-skills topics (e.g., financial literacy, educational and employment roadmaps, preparing for a doctor’s visit, knowing your family health history, understanding health insurance, and other topics). For example, participants could receive support to enroll in California’s Medicaid program or the Supplemental Nutrition Assistance Program or support in identifying community colleges or other educational institutions that met their interests and needs (e.g., type/level, location, financial aid, class scheduling). Participants also often received job-readiness supports (e.g., resume development, interviewing skills), were linked to employers supportive of reentrants.

The Health Fellowship was implemented by Health Coaches who were trained Marriage and Family Therapists. Health Coaches implemented a structured psychoeducational curriculum that draws on cognitive behavioral therapy, motivational interviewing, narrative therapy, and coaching techniques (Ojeda et al., 2022; Rollnick & Miller, 1995; Semmler & Williams, 2000; Wenzel, 2017). Service Navigators and Health Coaches assisted young adult reentrants in strengthening their executive functioning through skill-building vis-a-vis goal setting, time management, and building interpersonal communication skills and relationships, including conflict management using strategies such as motivational interviewing, role playing, and problem solving.

### Data Collection

#### Conceptual Model and Measures

Participants completed interviewer-administered surveys at baseline and 6 months post-enrollment; response sets are described in Table 1 Per the Gelberg-Andersen Behavioral Model of Service Utilization, *predisposing* factors are demographic factors, including age, gender, race/ethnicity, and educational attainment (Gelberg et al., 2000; Jones, 2017; Kulkarni et al., 2010; Phillips et al., 1998; Stein et al., 2007; U.S. Centers for Disease Controld and Prevention, 2015; U.S. Department of Veteran’s Affairs, 2016). *Enabling* variables are defined as health-supporting resources (e.g., marital status, employment status, food security, governmental identification, housing) and *need* factors included participants’ self-rated health (Aday, 1993; Andersen, 1995; U.S. Centers for Disease Controld and Prevention, 2015; U.S. Department of Veteran’s Affairs, 2016). Food security was assessed by asking participants: “In the past month, were you ever hungry but didn’t eat because there wasn’t enough money for food?” (response set: yes/no; Coleman-Jensen et al., 2014). The investigators developed items to assess possession of government identification and current participation in community services.

**Table 1.** Characteristics of Young Adult Probationers Ages 18 to 26 at Baseline and 6 Month Follow-Up (*n* = 66), Southern California, 2017 to 2021.

Variables	% or Mean at baseline	% or Mean at 6 months	*p*-Value
Predisposing factors			
Age			
Mean in years	21.9	22.4	.108
Gender			
Male	79%		N/A
Female	21%		
Race/ethnicity			
White, non-Latino (NL)	14%		N/A
Black/African American (NL)	29%		
Latino/Hispanic	47%		
Other race/ethnicity	11%		
High school/GED complete			
No	41%	38%	.722
Yes	59%	62%	
Enabling factors			
In a relationship/married			
No	77%	74%	.685
Yes	23%	26%	
Working full or part time			
No	65%	38%	.002
Yes	35%	62%	
Food insecurity			
No	68%	82%	.070
Yes	32%	18%	
Has any government-issued identification			
No	5%	0%	.122
Yes	95%	100%	
Housing: Own or pay rent for housing			
No	85%	73%	.089
Yes	15%	27%	
Receiving any other services/programming at enrollment (asked only at baseline)			
No	65%		N/A
Yes	34%		
Also			
Self-rated fair/poor health (vs. excellent, very good, or good)			
No	79%	80%	.829
Yes	21%	20%	

This study defines access to healthcare by analyzing the following variables: current health insurance coverage status, usual place of care, and use of healthcare in the past 6 months To further contextualize participants’ healthcare use experiences, we also report source of coverage, type of healthcare facility where care is usually received, any current unmet physical healthcare needs and types of services needed (U.S. Census Bureau, 2012; U.S. Centers for Disease Controld and Prevention, 2015). We created a continuous variable describing the number of unmet needs and the reasons for unmet needs. These data are shown in Table 2.

**Table 2.** Changes in Access to Care Between Baseline and 6-Month Follow-Up (*N* = 66) Among Young Adult Probationers Ages 18 to 26, Southern California, 2017 to 2021.

Access to care		Baseline (%)	6 month %	*p*-Value
Health insurance indicators				
Health insurance status	Not insured/don’t know	36%	12%	.001
	Insured	64%	88%	
Health insurance type	Not insured/don’t know	36%	12%	.012
	Medicaid (Medi-Cal)	50%	68%	
	Family member	11%	12%	
	Employer-based	0%	2%	
	Purchased/marketplace	0%	2%	
	Other insurance plan type	3%	5%	
Regular source of care				
Has a regular source of care	There is no place/don’t know	45%	30%	.073
	Yes, there was one or more than one place	55%	70%	
Regular source of care facility type	None	45%	30%	.092
	Doctor	14%	18%	
	Clinic	35%	44%	
	Urgent care/emergency room	5%	3%	
	Family member	0%	3%	
	Other	2%	2%	
Recent doctor/health visit				
Had a doctor/health visit in the past 6 months	No	64%	29%	.000
	Yes	36%	71%	
Unmet physical needs				
Any unmet physical health needs	No	56%	74%	.003
	Yes	44%	26%	
Number of unmet physical health needs (*M*)		1.0	0.3	.001
Unmet physical health needs	Dental care	29%	12%	.018
	Routine check-up	21%	6%	.010
	Vision care	18%	8%	.069
	Referral to a specialist (dermatologist, podiatrist, etc.)	12%	3%	.048
	Diagnostic test (MRI, CAT scan, X-ray, mammogram, etc.)	8%	2%	.104
	Prescription medication	6%	3%	.340
	Lab work (e.g., blood work, urinalysis)	3%	0%	.248
	Emergency care	2%	0%	.500
	Treatment for chronic issue	2%	0%	.500
	Other health need	2%	0%	.500
Reasons for unmet health needs at baseline (*n* = 29)	Could not afford it	48%		N/A
	No insurance	34%		
	Incarceration or justice system activity	24%		
	No transportation	17%		
	Too tired/lacked energy	10%		
	Forgot appointments	7%		
	Other reason	7%		
	Doctor did not take my insurance	3%		
	Physical health or disability	0%		
	Too long of a wait	0%		

#### Quantitative Data Analysis

Quantitative baseline and 6-month follow-up survey data were analyzed using STATA version 15 statistical software. As noted in the Limitations (below), the study experienced loss-to-follow up due to diverse circumstances (e.g., mobility, declining to complete the survey, COVID-19-pandemic onset). The authors conducted a sensitivity analysis comparing baseline characteristics (Table 1) of participants who did (*n* = 66) and did not (*n* = 73) complete the 6-month follow-up survey; no statistically significant differences between the groups were identified (data not shown; available on request). Descriptive statistics (i.e., means, frequencies) were calculated for predisposing, enabling, and need variables (Tables 1 and 2). Chi-square or Fisher’s exact tests were used to identify statistically significant (i.e., *p* < .05, *p* < .01, *p* < .001) changes between baseline and follow-up in dichotomous access to care variables (Table 2).

#### Analysis of Participant Satisfaction Text Data

Participants were invited to voluntarily complete an anonymous electronic Client Satisfaction Survey at 3 and 6 months which included the following open-ended question: (1) “*What changes have you noticed in yourself (or have others noticed in you) as a result of participating in the UCSD RELINK program?*” We used the “Coding Consensus, Co-occurrence, and Comparison” approach, based on grounded theory techniques, to analyze responses (Glaser et al., 1968; Willms et al., 1990). Authors [SHV, VO] used Microsoft Excel to analyze brief text responses (e.g., words, phrases) to generate main themes, their prevalence, and illustrative quotes (see Table 3).

**Table 3.** Major Themes Emergent from Participant Satisfaction Survey Open-Ended Question Responses Among Young Adult Probationers Ages 18 to 26 Enrolled Between May 2017 and September 2019, San Diego, CA (*n* = 42).

Themes	Illustrative quotes
Increased confidence and self-efficacy (40%, *n* = 17)	**• I feel more confident in myself in achieving more things in life. I really appreciate the staff here this was a very helpful program. I can say that I’ve picked up many life skills I’ve never been taught in my life.** I have noticed more awareness in myself as well as feeling confident about what to implement for certain situations and overall I feel a sense of peace. I am definitely more assertive of myself and less insecure about what others think. I feel more focused on my goals and look forward to achieving them. • Confidence and resilience. They help with general stuff which leads to a sense of relief and empowerment. **• I know about my health care life. . . [they] helped me by making concepts and information clearer to me so I can [make] better, more informed decisions**
Improved organizational skills and goal setting (33%, *n* = 14)	**• I know what my responsibilities are and how to manage my work, school, and life to not wear myself out. I have more understanding about my body’s health importance.** • I’ve notice myself going to a better direction, my emotions got more positive and was able to grasp a better understanding of my goals. • I have paid more attention to my needs in life and would like to start being in control of my life and the things I need to go through life.
Increased motivation and focus (29%, *n* = 12)	**• My mind and focus is better concentrated on the goals I have set for myself. I have a clearer plan for my future, health is important.** • Staying committed to my words and goal that I will achieve in my life. • That I’m more focused and carry myself better with improved posture. I’m more mindful about my decision making. **• [They helped] me understand more about health plans and they are helping me to be more productive in my life and to be a better person in life.**
Improved communication and interpersonal skills (26%, *n* = 11)	• I believe that this was a very helpful program for me. I can say I am a better different mother and citizen. I love the new changes I have applied to my life. **I have learned coping skills in how to deal with different situations in my daily life from how to communicate with my peers and family to how to manage stress, time, health, and other aspects that come my way. Thank You UCSD RELINK for teaching me something I never grew up with and now can move forward and teach to others.** • A better sense of understanding different views of a situation that involves confrontation. And just better communication skills in general. Starting to listen better, making eye contact at all times. Learning how to let issues go instead of stressing over the issues. Better at solving problems. Dealing with anger I tend to take time and practice my breathing.
Happier and more positive (24%, *n* = 10)	• I’m happier. • I reached my goals and I’m more positive and motivated and happy. Nothing is impossible. • I love myself again. I feel hope in my heart.
Improved self control and impulse control (22%, *n* = 9)	• Less stress, how to control myself, how to be responsible, getting along better with my girlfriend. • PATIENCE. I have been able to slow down and not be so rash. Let my decisions become options and not make decisions off one sided thinking. • I don’t react to everything right away anymore • [I’m] starting to listen better, making eye contact at all times. Learning how to let issues go instead of stressing over the issues. Better at solving problems. Dealing with anger—I tend to take time and practice my breathing.
Decreased stress and anxiety (20%, *n* = 8)	• I can say I don’t allow myself to get over stressed now with the few exercises I have taken. I can relax and find a solution without going crazy I’m calm and more responsible for my actions

*Note*. The bolded text refers to responses which directly mention health and healthcare topics.

## Results

### Participant Characteristics

Table 1 shows predisposing, enabling and need factors at baseline and 6 months follow-up. Most participants were male (79%) and had a mean age of 22 years. Nearly half were Latino (47%), 29% were Black/African American, 14% were non-Latino white and 11% identified as other race/ethnicity. At enrollment, less than two-thirds (59%) had completed high school or a high school equivalency test. About one in four participants was in a relationship or married, one third (35%) were employed, and one third (32%) reported being hungry in the past month and lacking money to buy food. Nearly all participants (95%) reported having at least one government identification. Just 15% of participants paid rent or owned their housing whereas the remainder were unstably sheltered, staying with friends/family, or in transitional housing. Importantly, only 34% reported being enrolled in other services or programs at baseline. One in five (21%) participants reported being in fair/poor health. At 6-month follow-up the proportion of participants working full- or part time improved significantly, rising from 35% at baseline to 62% at 6 months (*p* = .002). No other predisposing, enabling or need measures exhibited statistically significant changes over time.

### Access to Healthcare

Table 2 shows access to healthcare utilization outcomes between baseline and 6-month follow-up. The proportion of participants with health insurance coverage increased from 64% at baseline to 88% at follow-up (*p* < .001). The percent of participants insured via Medicaid (i.e., Medi-Cal in California), a public source of coverage, rose from 50% at baseline to 68% at follow-up. Having insurance through a family member was the second most common insurance type (11% at baseline to 12% at follow-up). A minority of participants had privately purchased, marketplace or employer-based insurance. The proportion of participants who reported at least one regular source of care increased from 55% at baseline to 70% at 6-months; this change was non-significant (*p* = .073; Table 2). The proportion of participants seeking care at a clinic (baseline: 35%–44% at 6-months) or doctor’s office (14% at baseline to 18% at follow-up) rose over time, though these changes were not statistically significant. The proportion of participants seeking healthcare services in the past 6 months increased from 36% at baseline to 71% at follow-up (*p* < .000). The proportion of participants reporting any unmet health needs at follow-up decreased significantly from 44% at baseline to 26% at follow-up (*p* = .003). The proportion of participants with unmet dental care needs decreased significantly (baseline: 29%–12% at 6-months, *p* = .018), as did the proportion of participants needing routine check-ups (baseline: 21%–6% at follow-up, *p* = .010) or a referral to a specialist (baseline: 12%–3% at follow-up, *p* = .048). Changes in other needs were non-significant. At baseline, the reasons most often given for having unmet healthcare needs (*n* = 29) included being unable to afford services (48%), lacking insurance (34%), being incarcerated or otherwise involved in the justice system (24%), or lacking transportation to attend appointments (17%).

#### Participants’ Self-reported Changes

Qualitative response data from participant satisfaction surveys revealed several themes, and these are presented alongside illustrative quotes where participants explicitly mention health and healthcare (see bolded font). Table 3 shows that 40% of participants reported increased confidence and self-efficacy, 34% reported improved executive functioning, organizational skills, and goal setting, 29% identified increased motivation and focus, 26% reported improvements in communication and interpersonal relationship skills, 24% were happier and more positive, 22% reported improved self-control and impulse control, and 20% identified reduced stress and anxiety levels due to program participation.

## Discussion

Adult reentrants, including young adults, often have complex needs that require multipronged interventions to improve healthcare access, health literacy, and the underlying social determinants of health (e.g., housing, education, employment, food security; Dumont et al., 2013; Hawks et al., 2020). Service Navigation and Health Coaching are gaining traction as strategies to address healthcare access challenges, particularly when individuals must coordinate across diverse social and healthcare service systems (Sanci et al., 2019). Yet, health focused reentry programs that integrate both Health Coaching *and* Service Navigation for *young adult reentrants* are scarce and critically needed to address long-term well-being. This pilot mixed-methods study addresses these gaps. Results revealed that providing Health Coaching and Service Navigation *in tandem* supported young adult probationers’ access to healthcare services (i.e., health insurance coverage, recent doctor visit) and reduced some unmet healthcare needs. Some participants reported enhanced knowledge, skills, and executive functioning (Gilbert & Burgess, 2008). The program also strengthened participants’ ties to local institutions that can support well-being. Further long-term exploration of the impacts of this intervention are needed to determine if gains observed in this study are retained over the long-term and whether similar outcomes are observed among other reentrant subgroups.

In the U.S., lack of health insurance coverage can limit individuals’ ability to offset the financial costs of obtaining preventive or therapeutic healthcare services in a timely manner (Yearby, 2018). While the United States’ Affordable Care Act has expanded access to health insurance coverage, not all states have expanded Medicaid coverage for low-income persons and racial and ethnic minorities continue to be over-represented among the uninsured (Winkelman et al., 2016). Significantly, in 2023, California became the first state in the U.S. to expand Medicaid health insurance coverage up to 90 days *prior* to reentry through a new program called “California Advancing and Innovating Medi-Cal” (i.e., CalAIM). This program seeks to enhance continuity in healthcare services, medications, and programming (California Department of Health Care Services, 2023a). This new policy and programmatic approach stand to decrease cost-related disparities in healthcare access among formerly incarcerated persons. However, results from this study suggests that efforts to connect reentrants to healthcare providers in their communities, including through provision of transportation, will likely still be needed to overcome logistical barriers. Data from this study indicate that reentrants can also benefit from tailored support, such as through Service Navigation, in order to address individuals’ unique barriers (e.g., scheduling, doctor visit preparation, self-advocacy) to healthcare utilization.

Young adult participants reported gains in insurance, thus likely contributing to increased use of health services over the study period. Participants also reported a decrease in selected unmet needs, including preventive services, access to specialists, and dental care. Without access to preventive services, individuals’ health can deteriorate into conditions and illnesses that are more costly to treat or which have long-term consequences (e.g., disability). This pilot study’s findings suggest that concerted efforts to eliminate barriers to healthcare access among young adult reentrants are critically needed. Individualized Service Navigation and Health Coaching services may assist young adults who lack natural mentors by providing the mentorship which can help them build life skills and executive functioning skills needed to engage with providers (e.g., time management, transportation planning, preparing for a doctor visit). Service Navigation can aid young adults identify critically needed resources to facilitate service utilization (e.g., enrolling in insurance, identifying a regular source of care, scheduling doctor visits). Peers with lived experience play an important role in mental health service utilization for young adults as they can serve as mentors, role models, and they may also connect with individuals from similar racial or ethnic backgrounds (Hiller-Venegas et al., 2022). Navigation support can be provided by peer coaches who have lived experience and credibility in the eyes of their clients as well as personal experience in navigating community services (Reingle Gonzalez et al., 2019) though specialized training, as noted in this study, can be an important component of setting peers up for success in this role (Wang et al., 2012).

### Limitations

Findings from this pilot study should be considered in light of the following factors. The study aimed to determine the feasibility and acceptability of a novel approach (i.e., coaching curriculum, didactic service navigation materials, pairing of Service Navigation and Health Coaching). This study is based on a convenience sample of young adult probationers; thus, individuals who may have benefited from the program but did not approach the study or were not at the recruitment sites may not be represented. The anonymous participant satisfaction survey was optional and not all participants provided feedback and the onset of the COVID-19 pandemic interrupted program satisfaction survey data collection. While qualitative studies are often characterized by smaller sample sizes, this study reported data from 42 participants, which is a larger sample and results can aid the researchers explore the impact of the intervention in future studies (Fugard & Potts, 2015). Several factors contributed to a smaller sample at 6-months, including declining to complete the survey, loss of contact with the study due to mobility or loss of a cell phone, or reincarceration. Notably, the sensitivity analysis did not demonstrate any differences between participants who did and did not complete the 6-month follow-up survey. Funders should consider including survey compensation as an allowable project expense to improve evaluation activities and data quality. Despite these considerations, the study makes an important contribution regarding the nature of support needed by young adult probationers who can benefit from stronger healthcare access. The resulting data have implications for broader strategies to eliminate disparities in health status and access to care among reentrants and other diverse and adjacent population-subgroups (e.g., unhoused persons, persons seeking employment).

## Conclusions

This study corroborates existing literature and finds that even young adult reentrants face multiple co-occurring challenges to engaging with health-supporting services and securing and stabilizing the social determinants of health (e.g., education, employment, income, housing, food security; Hawks et al., 2020; Kulkarni et al., 2010). This pilot study’s qualitative findings suggest that a team-based coaching-based intervention that builds knowledge, skills, self-confidence, and ties to community resources and institutions can foster human and social capital among young adult reentrants and this strategy is both acceptable and feasible. Future research can examine whether these mechanisms of change may also help to reduce disparities in health status and healthcare access among reentrants (Yearby, 2018). Longitudinal studies are needed to determine the long-term impacts of interventions such as this one. Critically, the participant satisfaction survey data suggest that this intervention’s structured coaching approach helped young adults build hard and soft skills across multiple domains as they transition to independence. Studies involving young adults living with mental illness suggest that peer providers are effective and tailored mentorship, problem-solving, and social support, including delivery of resources and emotional support, is often needed and welcome by young adults (Hiller-Venegas et al., 2022). Policymakers and program funders should consider expanding the scope of services available to young adult reentrants since, as a group, they are in ongoing development and may have unique needs. Significantly, young adults are at an important transition point between childhood and adulthood and intervening during this period may favorably impact young adults’ health, social and economic trajectories over the life course; this merits further research.
